# The Role of Mitochondrial Mutations in Chronification of Inflammation: Hypothesis and Overview of Own Data

**DOI:** 10.3390/life12081153

**Published:** 2022-07-29

**Authors:** Alexander N. Orekhov, Nikita G. Nikiforov, Andrey V. Omelchenko, Vasily V. Sinyov, Igor A. Sobenin, Andrey Y. Vinokurov, Varvara A. Orekhova

**Affiliations:** 1 Institute for Atherosclerosis Research, Osennyaya Street 4-1-207, 121609 Moscow, Russia; 2 Laboratory of Angiopathology, The Institute of General Pathology and Pathophysiology, 8 Baltiyskaya Street, 125315 Moscow, Russia; nikiforov.mipt@googlemail.com (N.G.N.); omi@bk.ru (A.V.O.); 3 Laboratory of Medical Genetics, Institute of Experimental Cardiology, National Medical Research Center of Cardiology, 121552 Moscow, Russia; st.rada201@gmail.com (V.V.S.); igor.sobenin@gmail.com (I.A.S.); 4 Cell Physiology & Pathology Laboratory of R&D Center of Biomedical Photonics, Orel State University, 95 Komsomolskaya Street, 302026 Orel, Russia; tolmach_88@mail.ru

**Keywords:** inflammation, mitophagy, mtDNA mutation, atherosclerosis

## Abstract

Chronic human diseases, especially age-related disorders, are often associated with chronic inflammation. It is currently not entirely clear what factors are responsible for the sterile inflammatory process becoming chronic in affected tissues. This process implies impairment of the normal resolution of the inflammatory response, when pro-inflammatory cytokine production ceases and tissue repair process begins. The important role of the mitochondria in the correct functioning of innate immune cells is currently well recognized, with mitochondrial signals being an important component of the inflammatory response regulation. In this work, we propose a hypothesis according to which mitochondrial DNA (mtDNA) mutations may play a key role in rendering certain cells prone to prolonged pro-inflammatory activation, therefore contributing to chronification of inflammation. The affected cells become sites of constant pro-inflammatory stimulation. The study of the distribution of atherosclerotic lesions on the surface of the arterial wall samples obtained from deceased patients revealed a focal distribution of lesions corresponding to the distribution of cells with altered morphology that are affected by mtDNA mutations. These observations support the proposed hypothesis and encourage further studies.

## 1. Introduction

An acute inflammatory reaction mediated by the innate immune system is a normal tissue response to a pathogen or injury. Under normal conditions, the inflammatory response resolves within a few hours or days depending on the injury scope, followed by the restoration of tissue structure and function. However, this process of normal immune response can be violated in a way that the resolution of inflammation is delayed or incomplete, leading to the development of a chronic inflammatory process. Such chronification of inflammation was shown to be associated with various human disorders. Human diseases that involve chronic inflammation account for up to 80% of total morbidity and mortality worldwide. Moreover, in high-income countries, the ageing population is increasingly affected by age-associated disorders, many of them having chronic inflammation as part of their pathogenesis. The term “inflammaging” was coined to distinguish such disorders focusing on the inflammatory component of pathogenesis and the possibilities of anti-inflammatory treatment approaches [1]. Atherosclerosis is one of the most common diseases associated with chronic inflammation, which accounts for a large amount of global mortality due to the cardiovascular events it provokes. In industrialized countries, atherosclerosis represents a major challenge to the healthcare system [2]. Chronic inflammation plays an important role at all stages of atherosclerosis progression: from the early events to the formation of unstable plaques, plaque rupture and thrombotic events [3]. Chronification of local inflammation in the arterial wall is, therefore, a crucial event in the pathogenesis of the disease.

**Figure 1:**
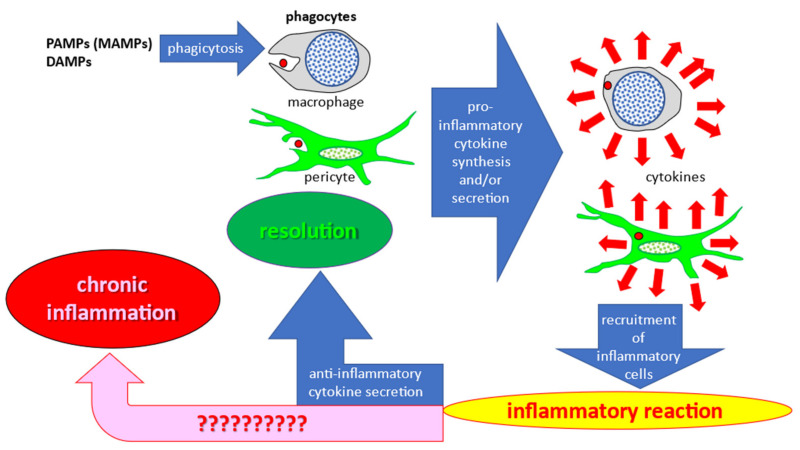
Schematic overview of the inflammatory reaction and chronification of inflammation in the arterial wall.

Recent studies have improved our knowledge of cellular mechanisms of inflammatory response. Pathogens, such as invading bacteria or damaged host tissues and cells, release pathogen-associated molecular pattern (PAMP) and damage-associated molecular pattern (DAMP) molecules, respectively. Once released in the extracellular space, these molecules activate the phagocytic activity of innate immune cells. Tissue macrophages are the primary phagocytic cells responsible for clearance of foreign objects (debris and pathogens) that initiate the immune response upon injury and/or infection. However, in the arterial wall intima, this function can also be performed by pericyte-like cells populating the subendothelial layer [4]. Phagocytosis stimulates the synthesis and/or secretion of pro-inflammatory cytokines, which act as a signal for the recruitment of immunocompetent blood cells. Hematogenous immunocompetent cells migrate to the developing inflammation site and actively participate in the immune response by phagocytosis and stimulation of other cells through pro-inflammatory cytokine secretion. However, this process normally ends with the release of anti-inflammatory cytokines that promote resolution of inflammation. Schematically, these processes are shown in Figure 1, with the chronic inflammation pathway shown by the corresponding arrow. Although a great amount of data has been accumulated on different aspects of the inflammatory process, the specific reasons leading to chronification of inflammation rather than the normal resolution process remain to be established.

In this review, we propose a hypothesis that mitochondrial dysfunction caused by mitochondrial DNA (mtDNA) mutations can be one of the causes leading to chronification of inflammation in atherosclerosis.

## 2. Presentation of the Hypothesis

Mitochondria are semi-autonomous organelles that appeared as a result of endosymbiosis of eukaryotic cells with ancient bacteria belonging to the Rickettsia genus 4 billion years ago [5,6]. Mitochondria still possess many bacterial features, including the circular genome that encodes for specific mitochondrial proteins. Given such similarities, is it possible that mitochondria can be mistakenly recognized as “invaders” by the innate immune system? Indeed, some authors admit the potential danger of mitochondria for cells that may mistakenly perceive them as bacteria that have got inside the cell [7]. In particular, it was recently reported that mitochondria can function as a source of DAMPs [7]. At the same time, mitochondria play an important role in the regulation of innate immunity in eukaryotic cells, and their correct functioning is indispensable for a proper innate immune response [8].

One of the features of mitochondrial semi-autonomy is the distinct organization of mtDNA and the mechanism of its replication and repair, which is different from that of nuclear DNA. MtDNA undergoes continuous cycles of replication and repair and exists in numerous copies, which allows for accumulation of mutations [9]. In fact, mtDNA mutations are one of the well-known hallmarks of aging, consistent with the fact that may diseases associated with mitochondrial dysfunction are age-related [10]. Although mtDNA has its functional repair mechanisms, the rate of mutagenesis in mtDNA is substantially higher than in nuclear DNA. That can be explained, among other factors, by the lack of protective histone packaging and the proximity of the mitochondrial genome to the sites of ROS generation on the mitochondrial membranes, which results in increased oxidative damage of mtDNA molecules [9]. Environmental factors and toxins are additional factors increasing mtDNA mutagenesis [10].

The host’s perception of the mitochondria as foreign agents requiring immune activation can be exacerbated by defective mitophagy—a mitochondria-specific autophagy that clears the defective or excessive mitochondria from the cell. Accumulating evidence highlights the role of mitophagy in immune response regulation and inflammation [11,12]. Dysfunctional mitochondria can be damaging to the cell through excessive generation of reactive oxygen species (ROS) that are normally produced in moderate quantities as by-products of mitochondrial energy production and quickly neutralized by cellular antioxidant systems. Defects in mitochondrial respiratory chain functioning abnormally increase ROS production, leading to oxidative stress. Moreover, defective and damaged mitochondria can trigger cell death through the release of pro-apoptotic factors to the cytoplasm. Mitophagy helps in mitigating these risks through isolation and controlled destruction of dysfunctional organelles. The protective role of mitophagy in cells in tissues has been illustrated by numerous studies. In particular, the microvascular ischemia-reperfusion injury (IRI) of microvascular endothelial cells is being actively studied in search for protective mechanisms that could alleviate the associated severe tissue damage. A study in a mouse model demonstrated that IRI led to upregulation of a nuclear receptor NR4A1, which was associated with abnormal mitochondrial fission, suppressed mitophagy and, as a consequence, increased endothelial injury [13]. At the same time, another study in transgenic mice demonstrated that inhibition of mitochondrial fission through Syk-Nox2-Drp1 signaling by the Bax inhibitor BI1 protected the endothelial cells during IRI [14]. These results highlight the crucial role of mitophagy in endothelial protection and open new possibilities for therapy development. A recent study demonstrated that the cardiovascular risk reduction effect of empagliflozin, an antidiabetic drug, is mediated by its protective action in microvascular endothelial cells, where it reduced the mitochondrial fission and improved mitochondrial respiration and oxidative stress during IRI [15].

Impaired clearance of dysfunctional mitochondria through mitophagy can have deleterious consequences for the cell. It leads to accumulation of mutant variants of mtDNA that are normally degraded together with dysfunctional organelles. Accumulation of dysfunctional organelles that generate excessive amounts of ROS because of impaired respiratory chain functioning lead to oxidative stress exacerbation and cellular damage. Deficient mitophagy can also result in the abnormal perception of damaged mitochondria as a pathogenic (bacterial) signal to trigger the innate immune response. This is primarily manifested by secretion of pro-inflammatory cytokines to recruit immunocompetent cells. As long as dysfunctional mitochondria remain inside the cell, the pro-inflammatory signal will continue without stopping. Conversely, mitophagy is known to have protective effects during the inflammation response in vascular endothelium. Under stress conditions, mitophagy and the mitochondrial unfolded protein response (UPR) act as protective mechanisms maintaining mitochondrial homeostasis and alleviating inflammatory myocardial injury. It was shown that the mitochondrial UPR is activated downstream of mitophagy, which plays a modulating role [16]. Therefore, improving mitophagy may prove a promising approach for protecting vascular endothelium from inflammation-associated damage. As mentioned above, the successful resolution of the inflammatory response is accompanied by the secretion of anti-inflammatory cytokines. In addition, the so-called “tolerance” of cells secreting cytokines contributes to the completion of the inflammatory reaction. Tolerance is manifested in the fact that, with repeated pro-inflammatory stimulation of cells, the secretion of cytokines is significantly reduced compared to the first stimulation [17,18,19]. Naturally, chronification of inflammation occurs when the inflammation resolution mechanisms are violated.

Previous studies by our group have demonstrated that atherosclerosis disrupts the pro-inflammatory response of monocytes and monocyte-derived macrophages [20]. At the same time, our group identified mutations in mtDNA (mitochondrial genome variants) associated with atherosclerosis in the aorta and blood cells of atherosclerotic patients [21]. We hypothesized that these mutations may be the cause of the impaired immune response of monocytes/macrophages.

## 3. Mitochondrial Mutations and Pro-Inflammatory Response in Atherosclerosis

**Figure 2:**
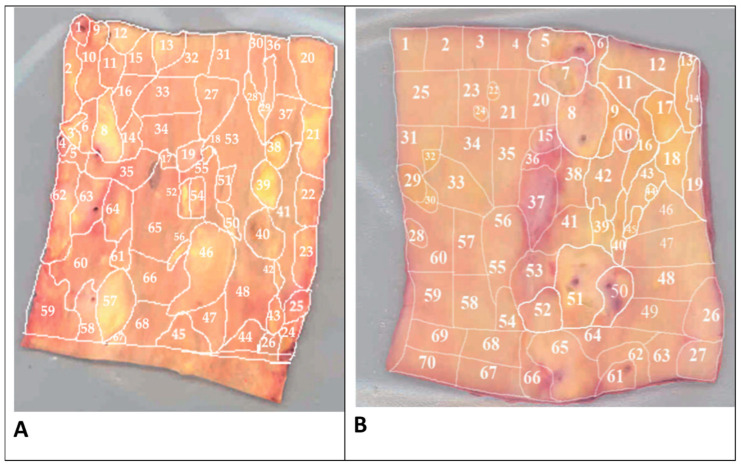
Morphologic mapping of the aorta tissue samples. Shown are two examples of morphologic mapping of the aortic wall samples. Segments of the vascular wall were divided according to morphological characteristics into 68 (**A**) and 70 (**B**) regions containing atherosclerotic lesions of varying severity (fatty infiltration, fatty streak, lipofibrous plaque, fibrous plaque) or intact tissue. These and other aorta samples were further analyzed for the mutational burden in mtDNA. (Reprinted with permission from [22], 2020, MDPI).

The involvement of mitochondrial mutations in atherosclerosis development is being actively studied. Indirect evidence of such involvement is the observed mosaic distribution of atherosclerotic lesions corresponding to the locations of cells bearing mitochondrial mutations in the arterial wall. The distribution of atherosclerotic lesions is never diffuse, but follows a local/focal pattern (Figure 2) [22].

**Figure 3:**
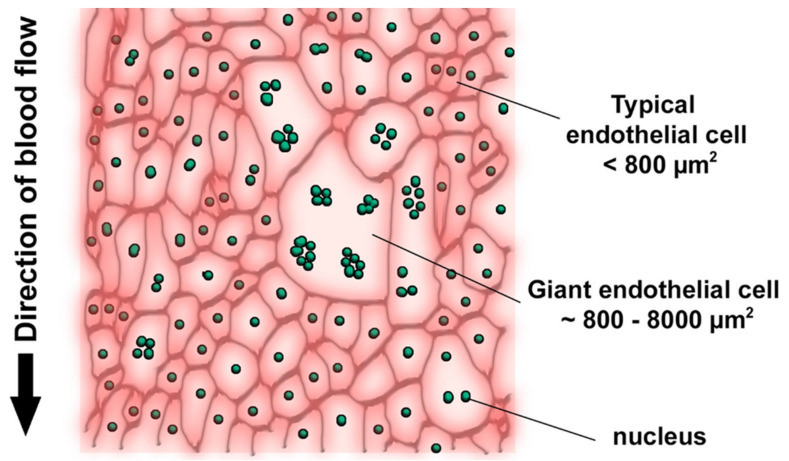
Schematic presentation of the heterogeneous endothelium of the adult human arterial wall. (Reprinted with permission from [27]. 2022, MDPI).

The observed mosaicism of atherosclerotic lesion distribution corresponds to that of the clusters of endothelial cells with impaired permeability. Human arterial endothelium consists of cells of various sizes [23,24,25,26]. Along with ordinary cells with typical morphology and size, the arterial endothelium contains giant multinucleated cells that usually form isolated clusters (Figure 3) [27].

It was recently reported that inflammatory cells as well as low-density lipoprotein, which is a source of fat in an atherosclerotic lesion, accumulate under such clusters [27]. Therefore, morphological observations suggest that the arterial wall may have local (focal) sites of inflammation associated with giant multinucleated endothelial cells. However, this suggestion does not clarify the reasons for inflammation chronification in atherosclerosis.

**Figure 4:**
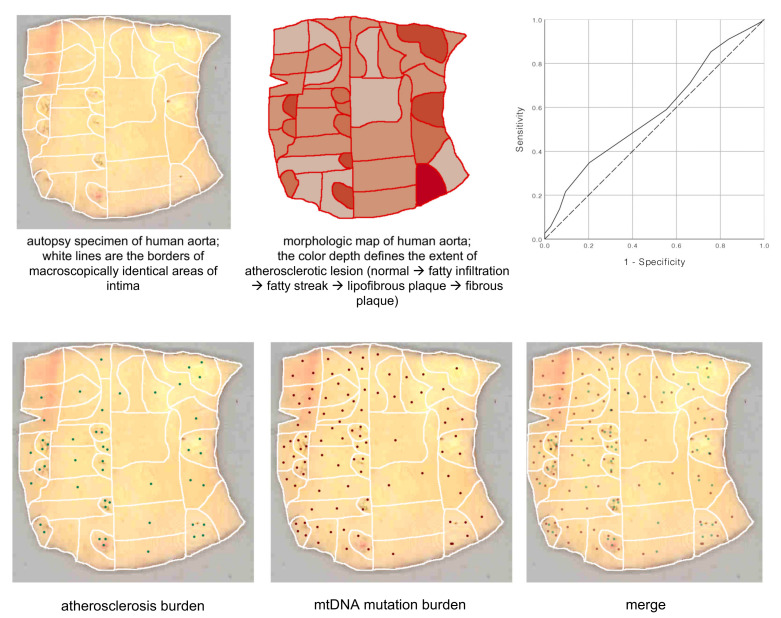
Mapping of colocalized mtDNA mutations and atherosclerotic lesions. (Reprinted with permission from [22], 2020, MDPI).

According to the working hypothesis of our group, the causes of chronification of inflammation may be mitochondrial dysfunction associated with mitochondrial mutations. This is indicated by the mosaic distribution of mtDNA mutations in the arterial intima (Figure 4) [22]. Mutations were identified in the unaffected intima and in various types of atherosclerotic lesions. Some of these mutations were associated with certain lesions and were distributed in the intima in a mosaic manner [21].

There was a significant correlation between the mtDNA mutation burden and atherosclerosis burden: r = 0.131, *p* = 0.034 (Spearman’s rho). The area under the ROC curve for the mtDNA mutation burden was 0.587 (95%CI 0.506–0.668 *p* = 0.041); the positive actual state was the presence of advanced atherosclerotic lesions (reprinted with permission from [22]).

To investigate the possible link between mtDNA mutations and the impaired pro-inflammatory response, lines of cybrids carrying atherosclerosis-associated mutations were created by our group. A cytoplasmic hybrid, or cybrid, is produced by repressing the cell line’s own mtDNA, followed by fusion of the whole cell with platelets containing mitochondria carrying the mutations of interest [28]. Using this method, we obtained several cell lines based on THP-1 macrophages and platelets from various atherosclerotic patients. These cybrid lines differed in the profile of atherosclerosis-associated mitochondrial mutations [29].

As mentioned above, the rate of mutagenesis (appearance of genetic variants) in mtDNA exceeds that in nuclear DNA. Mutations that are fatal for the cell are quickly eliminated together with the affected organelles if mitophagy is functional. Mutations that are not critical for the mitochondrial function are retained; however, it can be assumed that the combination of several mutations that appear in one mitochondrion can lead to mitochondrial dysfunction. This assumption is supported by the available data on the synergism of mitochondrial mutations in increasing the risk of atherosclerosis [30]. To identify a possible relationship between mitochondrial mutations and mitochondrial dysfunction, it is necessary to consider not only individual mutations, but also their combinations. Modern approaches and methods of bioinformatics make it possible to conduct such studies.

To study the pro-inflammatory activity of cybrid cells and their ability to form immune tolerance for successful resolution of inflammation, the secretion of pro-inflammatory cytokines (TNF, IL-1β, IL-6, IL-8 and CCL2) was evaluated upon a two-stage treatment of cells with a lipopolysaccharide (LPS) [31]. The first stimulation with the LPS (1 µg/mL for 24 h) was followed by the second stimulation (1 µg/mL for 4 h). It was shown that cybrid cell lines exhibited one of three types of pro-inflammatory response:

(1) Non-responders, which showed an extremely weak pro-inflammatory response to the LPS;
(2) Tolerant, which reacted to LPS but subsequently formed immune tolerance;
(3) Non-tolerant, which responded to LPS but did not form further tolerance.

Tolerant cells can be considered as normal, contributing to the successful resolution of inflammation. Non-tolerant cells are likely to be the cause of chronification of inflammation.

To reveal the relationship between mtDNA mutations and the pro-inflammatory activity of cells, the risk ratio (RR) value was calculated. The RR was calculated for each individual mutation as well as for all their possible combinations. The RR value reflected how many times more likely it is to detect a specific mutation or a combination of mutations in the considered group of cells compared to the probability of their detection in the other two groups. It was found that no individual mtDNA mutation was associated with the pro-inflammatory response. However, when studying combinations of mutations, two combinations were identified that were associated with a certain type of pro-inflammatory response in cybrids.

**Figure 5:**
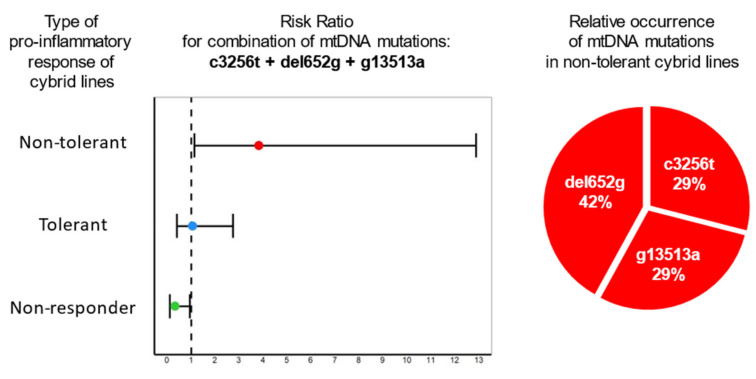
Association of the combination of mitochondrial mutations m.c3256t, m.del652g and m.g13513a with type of pro-inflammatory response of cybrid cell lines.

Figure 5 (left) shows the RR for the combination of mtDNA mutations m.c3256t, m.del652g and m.g13513a. Since the RR for the group of non-tolerant cells was greater than 1 and the area of the confidence interval did not cross 1, it can be concluded that this combination is associated with the group of non-tolerant cybrids (*p*-value < 0.001). At the same time, the RR for the group of non-responders was less than 1, and the area of the confidence interval also did not cross 1; that is, the probability of detecting a combination of the mutations m.c3256t, m.del652g and m.g13513a in this group is significantly low. In order to evaluate which of the three mutations could potentially have a greater contribution to the pro-inflammatory response, their occurrence relative to each other in a group of non-tolerant cells was assessed (Figure 5, right). It turned out that the m.del652g mutation was more common than the other two mutations m.c3256t and m.g13513a in the group of non-tolerant cybrids [31].

On the left is a graph of the risk ratio value, which reflects how many times more likely it is to detect a combination of mutations in the considered group of cells than in the other two groups. The horizontal lines show a 95% confidence interval. Cybrid cell lines were divided into three groups, differing in the type of pro-inflammatory activity: non-responder (exhibited an extremely weak pro-inflammatory response to LPS), tolerant (secreted pro-inflammatory cytokines in response to LPS, after which they formed tolerance to LPS) and non-tolerant (responded to LPS but did not further develop tolerance to LPS). On the right is the occurrence of each mutation mt.c3256t, m.del652g and m.g13513a relative to the other two in intolerant cybrid lines [31].

**Figure 6:**
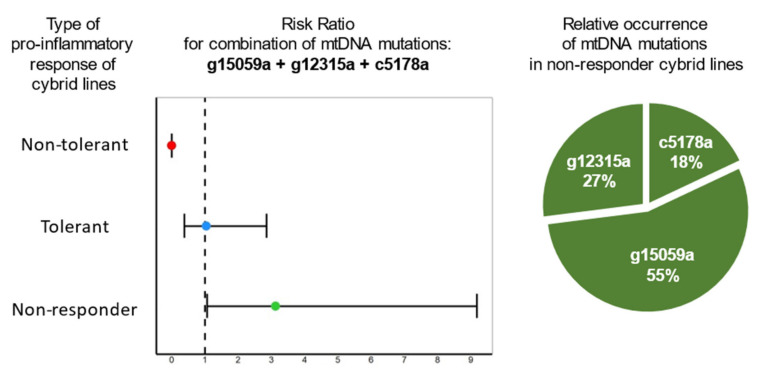
Association of the combination of mitochondrial mutations m.g15059a, m.g12315a and m.c5178a with type of pro-inflammatory response of cybrid cell lines [31].

Figure 6 (left) similarly shows the RR for the combination of mtDNA mutations m.g15059a, m.g12315a and m.c5178a, from which it follows that this combination may be associated with the group of non-responder cybrids. Similarly, the relative occurrence of each mutation in the non-responder group was calculated (Figure 6, right). It turned out that the m.g15059a mutation is more common than the other two studied mutations [31].

Therefore, our group revealed a combination of mtDNA mutations that may mediate the impairment of macrophage tolerance (the ability to reduce the pro-inflammatory response during the subsequent stimulations).

## 4. Mitophagy in Immunity

Playing a central role in metabolism, mitochondria are also key players in the regulation of the immune and inflammatory responses [32]. Several studies reported the immunogenic capabilities of dysfunctional mitochondria [7,33]. Mitochondrial dysfunction, and in particular, a mitophagy defect, is also associated with immune response disturbance [34]. Defective mitophagy can lead to excessive secretion of pro-inflammatory cytokines, therefore activating the innate immune response at the immune cell level. This contributes to pathological inflammation in autoimmune diseases [35].

Moreover, mitophagy may play a central role in sepsis [36]. Upon inhibition of mitophagy with pharmacological inhibitors, macrophages can cause an increase in macrophage migration inhibitory factor secretion and exacerbate inflammation [37]. On the other hand, excessive mitophagy can lead to macrophage apoptosis, which in turn further exacerbates the inflammatory response [38].

During viral infection, mitophagy has an anti-inflammatory effect [39,40,41]. Viral infection induces mitophagy to inhibit apoptosis and NLRP3-mediated inflammation through eliminating damaged mitochondria that can release ROS and pro-apoptotic factors that promote virus reproduction [42]. Mitophagy is involved in immune response regulation through the suppression of NLRP3 inflammasome activation, which induces the immune response to DAMPs and PAMPs [43]. In case of NLRP3 inflammasome activation by free mtDNA, elimination of mitochondria through mitophagy suppresses its hyperactivation and corresponding immune response [44].

Another safeguard against NLRP3 hyperactivation is the stress-induced sestrin2 protein [45]. In sestrin2-deficient mice, macrophages are characterized by hyperactivation of caspase-1 and increased secretion of IL-1β and IL-18. Upon stimulation with the LPS, sestrin2 promotes mitophagy, thereby preventing prolonged activation of the NLRP3 inflammasome and immune response stimulation [41].

Among the known inhibitors of excessive inflammatory response are PINK1 and Parkin proteins that act via the STING signaling pathway by preventing the release of mtDNA from dysfunctional mitochondria [45]. Transgenic mice deficient for PINK1 and Parkin are more susceptible to polymicrobial sepsis as compared to wild-type animals. However, the increased susceptibility of these knockout mice to sepsis was reduced with depletion of the NLRP3 inflammasome [46]. Mitophagy induced by PINK1/Parkin activation is also associated with the STING pathway in response to mtDNA released to the cytosol [45]. Thus, mitochondrial destruction triggers an inflammatory response not only through the NLRP3 inflammasome, but also through the cGAS-STING signaling pathway [47]. Deficiency of PINK1 and Parkin leads to increased STING activation [45]. Furthermore, mice deficient for these proteins upon depletion of STING show an impaired inflammatory response, therefore supporting the idea that PINK1 and Parkin can prevent mtDNA release, and thus, inhibit abnormally high inflammatory responses via the STING signaling pathway [45].

## 5. Mitophagy and mtDNA Mutations

The available data allow us to conclude that mitophagy is actively involved in innate immunity and pathological inflammation. It can be argued that normal mitophagy is essential for the correct resolution of inflammation. Conversely, disruption of mitophagy due to some defects can lead to chronification of inflammation. These ideas are confirmed by the results of experiments on cybrid lines of macrophages.

**Figure 7:**
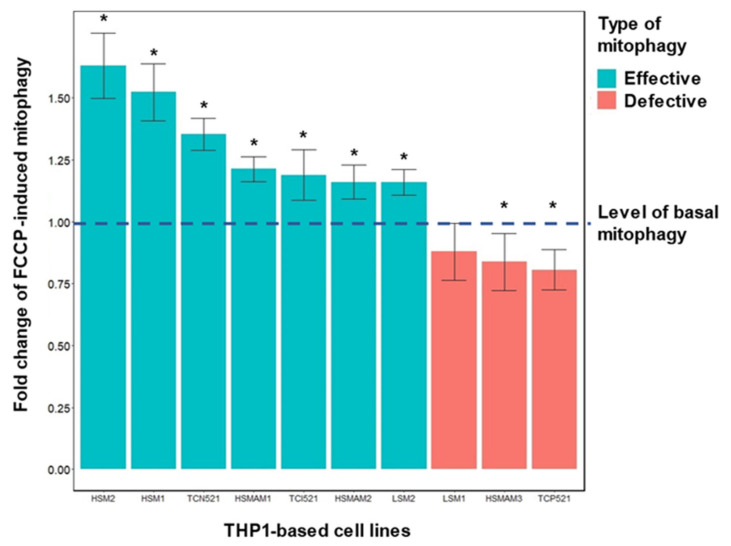
Induction of mitophagy with FCCP in cybrid lines carrying atherosclerosis-associated mtDNA mutations. Shown is the change in mitophagy under the influence of FCCP relative to the basal level of mitophagy in ten cybrid lines. Mitophagy was assessed as a 12 h colocalization of mitochondria and lysosomes, which were labeled with MitoTracker Green FM (200 nM) and LysoTracker Red DND-99 (50 nM), respectively. FCCP (2 μM) was used for stimulation of colocalization in the experiments with induced mitophagy. Confocal images were obtained using a Zeiss 900 confocal microscope equipped with a 63× oil immersion objective. MitoTracker Green fluorescence intensity was obtained with 488 nm excitation and 500–530 nm emission filter. The 561 nm excitation line and 566–700 nm emission filter were used for LysoTracker Red DND-99. Colocalization was calculated with ZEISS ZEN 3.1 (blue edition) software as the relative number of colocalized pixels in MitoTracker Green in relation to the total number of pixels above the threshold value. The basal level of mitophagy was taken as one and marked with a dotted line. Cybrid lines with efficient mitophagy, i.e., with increased intensity of mitophagy under the influence of FCCP, are represented in green. Cybrid lines with defective mitophagy, i.e., either not different from the basal level or below the basal level, are marked in red. An asterisk indicates significant differences between induced mitophagy and basal mitophagy, *p* < 0.05, according to the results of the Wilcoxon non-parametric paired test [31].

In one such experiment, 10 lines of cybrids carrying various mitochondrial mutations associated with atherosclerosis were investigated (Figure 7). Mitophagy was induced by two agents: FCCP (trifluoromethoxy carbonylcyanide phenylhydrazone) and pyruvate. When stimulated with FCCP at a low concentration (2 μM), PINK-dependent mitophagy is induced by mitochondrial membrane depolarization. In the case of sodium pyruvate, PINK-independent mitophagy is stimulated by slight acidification of the cytosol [48,49]. The results are presented in Figure 7.

In three out of ten cybrid lines, mitophagy was found to be defective when activated with FCCP (Figure 7). Cell lines with defective mitophagy included LSM1, HSMAM3 and TCP521. In the HSMAM3 and TCP521 lines, mitophagy under the influence of FCCP decreased below the basal level, i.e., an inversion of the FCCP effect was observed [31].

**Figure 8:**
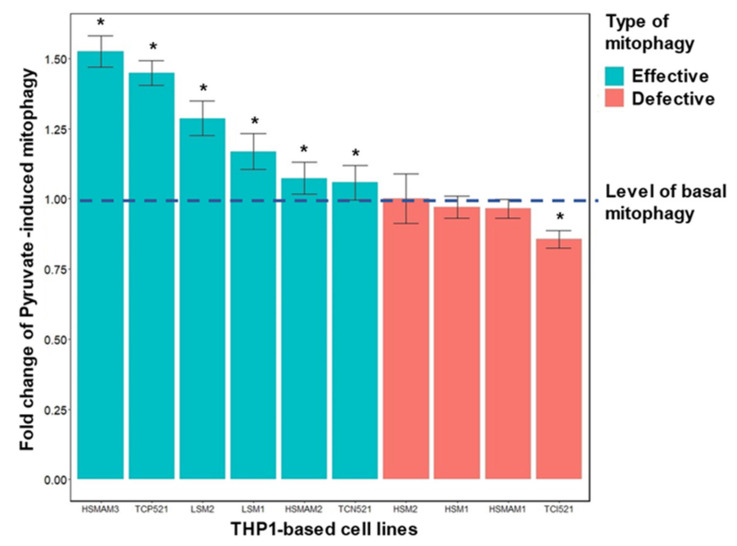
Induction of mitophagy with pyruvate in cybrid lines carrying atherosclerosis-associated mtDNA mutations. Shown is the change in mitophagy under the influence of pyruvate relative to the basal level of mitophagy in ten cybrid lines. The experiment procedure was the same as given in the description of Figure 7 with exception of using of sodium pyruvate (20 mM) for colocalization stimulation. The basal level of mitophagy was taken as one and marked with a dotted line. Cybrid lines with efficient mitophagy, i.e., with increased intensity of mitophagy under the influence of pyruvate, are represented in green. Cybrid lines with defective mitophagy, i.e., either not different from the basal level or below the basal level, are marked in red. An asterisk indicates significant differences between induced mitophagy and basal mitophagy, *p* < 0.05, according to the results of the Wilcoxon non-parametric paired test [31].

Upon induction of mitophagy with pyruvate, defective mitophagy was observed in four cybrid lines: HSM2, HSM1, HSMAM1 and TCI521 (Figure 8). Moreover, defective PINK-independent mitophagy induced by pyruvate was observed in cybrid lines that were different from the lines defective for PINK-dependent mitophagy induced by FCCP.

Using bioinformatics methods, a statistically significant relationship was revealed between the degree of mitophagy and presence of m.g12315a and m.g14846a mutations. It was found that only the m.g14846a mutation was associated with defective mitophagy. The m.g14846a mutation is located in the *MT-CYB* gene encoding for cytochrome B, a protein that is involved in the electron transport chain. Mutation m.g14846a results in glycine to serine substitution in position 34, thus affecting the intermediate transfer of electrons in the mitochondrial respiratory chain. Such substitution impairs the enzymatic function of cytochrome B, and is associated with mitochondrial myopathies [50,51].

**Figure 9:**
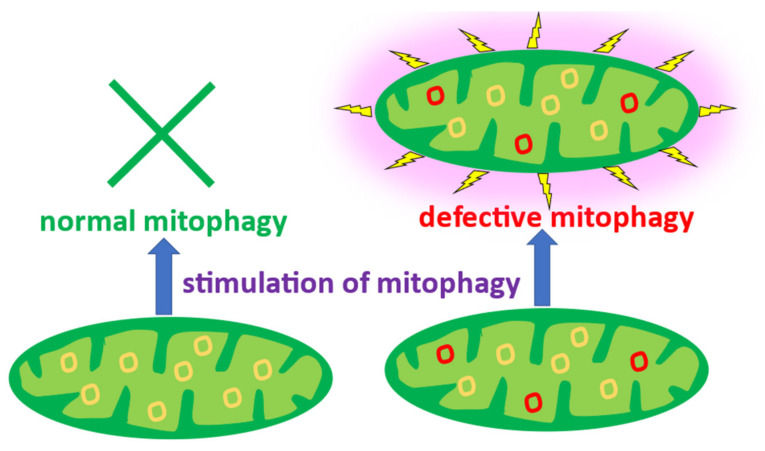
The hypothetical role of the mtDNA m.g14846a mutation in the formation of defective mitophagy that induces an excessive non-stop inflammatory response.

A mathematical model study indicated that an increase in heteroplasmy of the m.g14846a mutation by 1% leads to a decrease in mitophagy by 4%; that is, if the heteroplasmy of this mutation reaches 25%, mitophagy will be defective (Figure 9).

Mitochondria with defective mitophagy remain in the cell for a long time and are not subject to elimination. The dysfunctional organelles can be perceived as pathogens, triggering a non-resolving inflammatory response. This can be one of the mechanisms of chronification of the inflammatory response in human disorders.

It is noteworthy that there is a strong association between the m.g14846a mutation and defective mitophagy. No such relationship was observed between individual mitochondrial mutations and the impaired immune response. It was only possible to identify an association between a combination of several mutations and a decrease in tolerance to pro-inflammatory stimulation or with the absence of a pro-inflammatory response (Figure 5 and Figure 6). Various mechanisms, including various signaling pathways, may be involved in the disruption of the cellular pro-inflammatory response. The regulation of these pathways can involve both mitochondria with defective and normal mitophagy, as well as other processes that are not directly related to mitochondria and mitochondrial mutations. It can be assumed that mitochondrial mutations are involved in the disruption of the immune response in a complex way. This is confirmed by the revealed absence of association of defective mitophagy and the type of immune response of cybrids.

**Figure 10:**
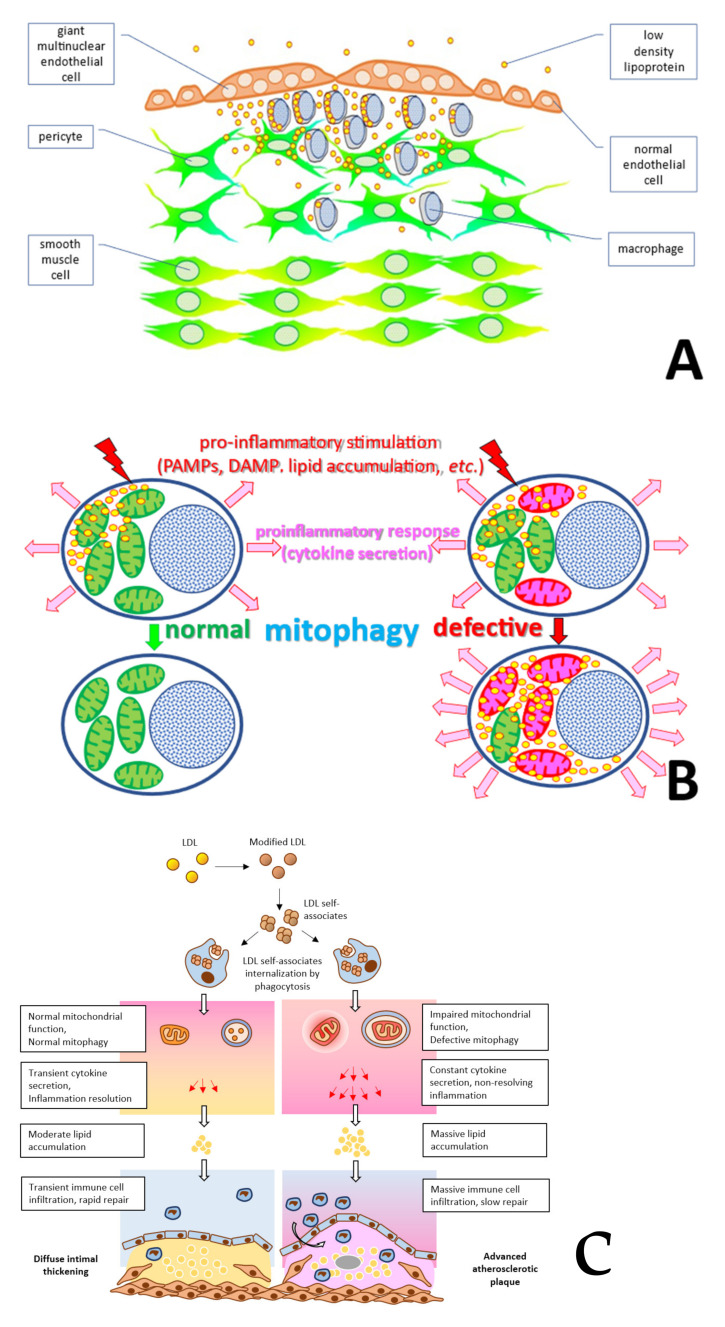
Proposed mechanisms of chronic inflammation in atherogenesis. (**A**) Accumulation of circulating immune cells and low-density lipoprotein (LDL) taking place preferentially in the areas enriched with giant multinucleated endothelial cells. (**B**) Pro-inflammatory signaling initiated in response to uptake of modified LDL can become persistent in presence of defective mitophagy. (**C**) Normal resolution (left) and chronification of inflammation (right) in the arterial wall lead alternatively either to diffuse thickening or to persistent inflammation and atherosclerotic plaque formation. (Panel C reprinted with permission from [27], 2020, MDPI).

The discussed observations allow the formulation of a hypothesis about the role of mitochondrial mutations in the chronification of inflammation. This hypothesis was already tested on the example of atherosclerosis (Figure 10) [27], but can prove useful in other chronic human diseases associated with inflammation.

Accumulation of circulating immune cells that differentiate into macrophages and modified low-density lipoprotein (LDL) can occur preferentially in the areas of the arterial wall enriched with giant multinucleated endothelial cells (Figure 10A). These areas become the sites of local (focal) inflammation. Modified LDL self-associates are taken up by macrophages and pericytes through phagocytosis, initiating the immune pro-inflammatory response and secretion of pro-inflammatory cytokines (Figure 10B). In turn, cytokines enhance the accumulation of lipoprotein and contribute to the development of cellular lipidosis. If mitophagy is functional, acute focal inflammation quickly ends with resolution leading to the formation of focal sites with increased tissue density. Since focal inflammation occurs regularly throughout life, diffuse thickening of arterial wall intima accumulates with age, without leading to atherosclerosis development (Figure 10C, left). However, if the cells involved in the pro-inflammatory response carry mitochondrial mutations causing defective mitophagy (Figure 10C, right), the pro-inflammatory response is increased, and focal inflammation does not end with a resolution and becomes chronic. This leads to a pronounced lipid accumulation in the affected sites, giving rise to an atherosclerotic lesion.

## 6. Conclusions

It was revealed that combinations of mtDNA mutations are associated with the impairment of macrophage tolerance (the ability to reduce the pro-inflammatory response during the subsequent stimulations). Numerous works have shown that mitochondrial dysfunctions, and in particular, mitophagy defects, are also associated with immune response disturbance. Defective mitophagy can lead to excessive secretion of pro-inflammatory cytokines, therefore activating the innate immune response at the immune cell level. This contributes to pathological inflammation in autoimmune diseases. Using bioinformatics methods, a statistically significant relationship was revealed between the degree of mitophagy and a panel of mtDNA mutations. It was found that a certain mutation is associated with defective mitophagy.

In this work we proposed a hypothesis that allows linking the mitochondrial mutations to chronification of the inflammatory process, which is relevant for various chronic human diseases. Mitochondrial mutations are characterized by the level of heteroplasmy, or proportion of mutated variant, in the mtDNA pool in a cell. Cells reaching a threshold level of heteroplasmy for certain mutations can have impaired mitophagy, thereby accumulating dysfunctional mitochondria that can lead to prolonged stimulation of the inflammatory reaction and leading to chronification of inflammation. In atherosclerosis, a well-known disease associated with chronic inflammation, the proposed hypothesis allowed for an explanation of the focal distribution of atherosclerotic plaques on the arterial wall surface. Further studies will continue the search for mtDNA mutations relevant for inflammation disturbances and will test mitochondria-targeting therapies for treatment of chronic inflammatory disorders.

## Data Availability

Not applicable.
